# Supplementary material for: Design of allosteric modulators that change GPCR G protein subtype selectivity
Source: Res Sq. 2024 Dec 11:rs.3.rs-5538058. Preprint. [Version 1] doi: 10.21203/rs.3.rs-5538058/v1 (PMC11661308; doi:10.21203/rs.3.rs-5538058/v1)
Supplement: Supplement 1 [file NIHPPRS5538058V1-supplement-1.pdf]

Supplementary Files

This is a list of supplementary files associated with this preprint. Click to download.

- SupplementaryMaterial1124Final.pdf
